# Action Representation in Patients with Bilateral Vestibular Impairments

**DOI:** 10.1371/journal.pone.0026764

**Published:** 2011-10-24

**Authors:** Laurent Demougeot, Michel Toupet, Christian Van Nechel, Charalambos Papaxanthis

**Affiliations:** 1 Université de Bourgogne, UFR STAPS, Dijon, France; 2 Institut National de la Santé et de la Recherche Médicale, Unité 887, Motricité et Plasticité, Dijon, France; 3 Centre d'Exploration Fonctionnelles Oto-Neurologique, Paris, France; 4 IRON, Institut de Recherche en Oto-Neurologie, Paris, France; 5 Unité Troubles de l'Equilibre et Vertiges, CHU Brugmann, Brussels, Belgium; 6 Unité de Neuro-Ophtalmologie, CHU Erasme, Brussels, Belgium; University of Bologna, Italy

## Abstract

During mental actions subjects feel themselves performing a movement without any corresponding motor output. Although broad information is available regarding the influence of central lesions on action representation, little is known about how peripheral damages affect mental events. In the current study, we investigated whether lack of vestibular information influences action representation. Twelve healthy adults and twelve patients with bilateral vestibular damage actually performed and mentally simulated walking and drawing. The locomotor paths implied one (first walking task) and four (second walking task) changes in the walking direction. In the drawing task, participants drew on a sheet of paper a path that was similar to that of the second walking task. We recorded and compared between the two groups the timing of actual and mental movements. We found significant temporal discrepancies between actual and mental walking movements in the group of patients. Conversely, drawing actual and drawing mental durations were similar. For the control group, an isochrony between mental and actual movements was observed for the three tasks. This result denotes an inconsistency between action representation and action execution following vestibular damage, which is specific to walking movements, and emphasizes the role of the vestibular system upon mental states of actions. This observation may have important clinical implications. During action planning vestibular patients may overestimate the capacity of their motor system (imaging faster, executing slower) with harmful consequences for their health.

## Introduction

Mental movement simulation or motor imagery is a state of mental rehearsal during which subjects internally replicate a movement (first-person perspective) without any corresponding motor output. It is now broadly admitted that mental actions are motor intentions and plans that are not overtly executed. Actual and mental actions rely on similar motor representations and activate common brain areas [1], [2], [3]. Notably, the parietal and prefrontal cortices, the supplementary motor area, the premotor and primary motor cortices, the basal ganglia and the cerebellum are activated during both actual and mental movements [1], [4]. In addition, autonomic activation increases proportionally to mental effort produced by subjects during imagined movements [5], [6], [7]. At the behavioural level, the analysis of the temporal aspects of mental actions is a potential tool to investigate similarities between movement production and its mental replication. In particular, mental actions retain the same temporal structure and follow the same motor rules (e.g., speed-accuracy trade-off) as their actual counterparts [8], [9], [10], [11], [12], [13], [14]. Timing information during mental simulation is the output of internal forward models, which are neural networks simulating the behaviour of the body and its interaction with the environment [15], [16], [17].

Timing inconsistencies between actual and mental movements has been used as a criterion to investigate the effects of neurological deficits on action representation. Several studies have reported that movement representation declines in patients with lesions in the parietal cortex [18], [19] and the cerebellum [20]. Furthermore, action representation is deteriorated in patients affected by stroke [21], Parkinson disease [22], and schizophrenia [23]. In addition, the capacity to mentally simulate motor actions progressively declines in healthy elderly people [24], [25], [26], [27]. Although broad information is available regarding the influence of central lesions on action representation, little is known about how peripheral damages affect mental events. In the current study, we investigated whether action representation was affected in patients with bilateral vestibular loss. We expected that mental actions would be deteriorated after vestibular diseases, because vestibular information is essential in mental transformation tasks. For instance, bilateral vestibular loss [28], caloric [29] and galvanic vestibular stimulation [30], as well as microgravity [31] strongly weakness spatial imagery, object mental rotations, and egocentric mental transformations. Neuroimaging studies also corroborate the premise that vestibular damage could influence action representation. In humans, vestibular projections reach several brain areas, such as the parieto-insular vestibular cortex, the somatosensory cortex, the area MST, the intraparietal sulcus, and the hippocampus (for a review, see [32]). Most of these areas are also activated when subjects imagine walking. For instance, Malouin and collaborators [33] have reported the activation of the precuneus and dorsal premotor cortex bilaterally, the left dorsolateral prefrontal cortex, the left inferior parietal lobule, and finally the right posterior cingulate cortex. These structures are part of a well-documented neural network [1] associated with visuo-spatial processing of motor actions in space, the planning of sequential movements and their motor simulation from a first person perspective.

In the current study, healthy adults and patients with bilateral vestibular loss actually and mentally performed two walking and one drawing task. We recorded and compared between the two groups the timing of actual and mental movements. We anticipated that vestibular damage should affect walking, but not drawing movements. As vestibular signals are important for balance and spatial orientation [34], [35], vestibular patients should actually walk slower than healthy adults. Furthermore, as vestibular information is essential for mental operations [28], [29], temporal differences between actual and mental walking movements should be observed in vestibular patients, but not in healthy adults.

## Materials and Methods

### Ethical statement

All participants gave their written informed consent prior to their inclusion in this study, which was carried out in accordance with legal requirements and international norms (Declaration of Helsinki, 1964). The protocol was approved by the Dijon Regional Ethics Committee.

### Participants

Twenty four adults voluntarily participated in the present study. They were divided into two groups: a control group of healthy participants (8 males and 4 females; mean age  = 43.4±7 years) and a group of patients (9 males and 3 females; mean age  = 50.3±14 years). Healthy participants were examined by a medical doctor and all had normal or corrected vision, and did not present any cognitive, neurological, or muscular disorders. Patients suffered from bilateral idiopathic loss of vestibular function (BILVF), but not from hearing loss or associated neurological symptoms. All patients had acquired their vestibular loss at least 5 years prior to this experiment (with either simultaneous or sequential onset of BILVF), and none complained of persistent oscillopsia. Bilateral impairments of vestibular function were assessed by a battery of functional tests before the inclusion of the patients in the experiment. Video-nystagmography with bi-thermal caloric irrigation was performed for the right and the left ear at both 44 °C and 30 °C. The Head Impulse Test was performed with an automatic sensitivity video camera system for each of the six canals. These tests clearly revealed bilateral dysfunctions of semi-circular canals for all patients. Furthermore, subjective visual vertical estimation and posturographic measures of steadiness also confirmed the absence of vestibular function for all patients. Participants received general information about the experimental procedures prior to the experiment, but none of them were informed about the specific hypothesis of the study.

### Motor tasks and Experimental protocol

**Figure 1:**
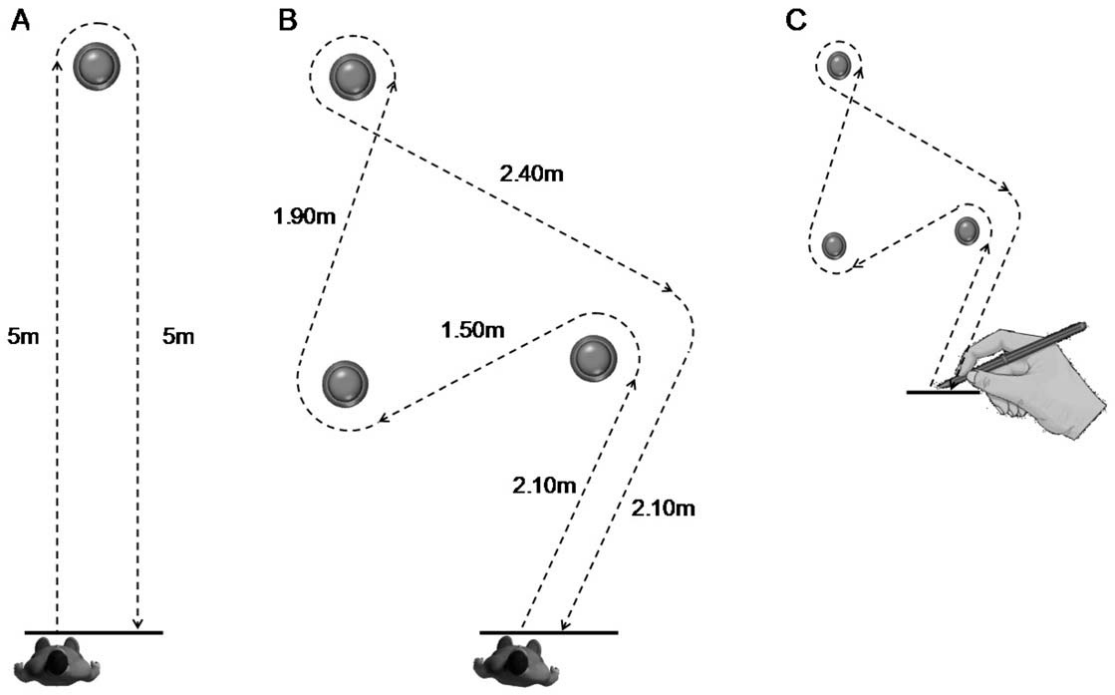
Motor tasks. (A) First walking task: the participants actually or mentally walked along a path which consisted of two straight lines and implied one change in the walking direction. (B) Second walking task: the participants actually or mentally walked along a path which consisted of five straight lines and implied four changes in the walking direction. (C) Drawing task, the participants had to draw on a sheet of paper a path that was similar to that of the second walking task. Plots, arrows and lines indicated the paths and the starting-finishing areas.

The experiments took place in a quiet room (8 m×6 m) which was illuminated with homogenous white light. Participants were requested to actually perform and to mentally simulate walking and drawing. In the first walking task, participants actually or mentally walked along a path which consisted of two straight lines and implied one change in the walking direction (Fig. 1A). In the second walking task, they actually or mentally walked along a path which consisted of five straight lines and implied four changes in the walking direction (Fig. 1B). Plots, arrows and lines were drawn on the ground to indicate the two paths and the starting-finishing areas. In the drawing task, participants had to draw on a sheet of paper a path that was similar to that of the second walking task (Fig. 1C). This task was mainly used as a control condition for the group of patients. Participants were requested to execute and to mentally simulate the motor tasks at a natural self-selected speed and with their eyes open in order to preserve their equilibrium during locomotion. It was specified to them that they must feel themselves performing the task (kinaesthetic imagery) rather than just visualizing (visual or external imagery). After the achievement of the experimental protocol, none of the participants reported difficulties to internally simulate the movements. In addition, they had verbally reported after each mental trial that they have completed the path from start to finish. All participants remained immobile during mental movements.

### Recording of actual and mental movement durations

Durations of actual and mental arm movements were recorded by means of an electronic stopwatch (temporal resolution 1 ms). The mental chronometry paradigm has already given reliable results in many behavioural studies [9], [12], [36], [37]. For the actual or the mental accomplishment of the walking tasks, the participants were standing upright in front of a line drawn on the ground. Their arms were hanging each side and their feet were parallel and slightly apart. They started the stopwatch (dominant hand) when they actually or mentally initiated their first step, and they stopped it when they actually or mentally finished their last step. One trial consisted of one repetition of the walking path. For the actual or the mental accomplishment of the drawing task, the participants sat on a chair in front of a table whose edge was aligned with their chest at the level of the diaphragm. For each trial, participants were presented with a plain sheet of paper (A4 format, placed at a distance of 10 cm from their chest level) on which we drew a template similar to the second walking path. The participants, holding a pencil in their right-dominant hand, were asked to trace or to imagine tracing through the template. They started the stopwatch (non-dominant hand) when they initiated the hand movement and they stopped it when they finished drawing. One trial consisted of two repetitions of the drawing template. Each participant accomplished 10 trials in each experimental condition (i.e. a total of 60 trials). Participants completed first the mental movements and then the actual movements, while motor tasks were carried out in a random order. A five minute interval separated the two execution modalities. Furthermore, when a participant performed 2 consecutive trials, he/she rested for ∼1 minute to avoid physical and mental fatigue. Participants did not have knowledge of their actual or mental temporal performances. In order to familiarize themselves with the experimental protocol, the participants performed one actual and one mental trial for each motor task before the experiment.

### Data and statistical analysis

For each participant, we calculated average duration of the ten trials in each experimental condition and verified that normal distribution was respected (Shapiro-Wilk test). We performed ANOVA with *group* (control, patients) as between-subjects factor and *execution mode* (actual, mental) and *task* (two walking and one drawing) as within-subjects factors. This analysis would mainly reveal whether actual and mental movement durations differed between the two groups.

We performed a complementary analysis across each group. Specifically, for the different motor tasks, we compared the temporal similarities between actual and mental actions to appreciate to what extent action representation is similar to action execution. When mental duration significantly differs from actual duration, one could argue that some aspects of movement execution are not included, or partially integrated, into action representation.

We calculated the relative index of mental/actual performance (rIP):
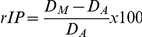



For each participant, rIP is defined as the difference between the average duration of mental movements (*D_M_* in the formula; n = 10) and the average duration of actual movements (*D_A_* in the formula; n = 10). In order to account for inter-individual differences in movement duration, we divided this value by the average actual movement duration (*D_A_*). The rIP index indicates whether subjects overestimate (positive values) or underestimate (negative values) the durations of mental movements with respect to actual movements. For instance, an index of 100% would indicate that the duration of mental movements is 2 times greater from that of actual movements. On the contrary, a near to zero index would indicate excellent mental performance, i.e., almost similar actual and mental movement durations.

Although interesting, this index could conceal discrepancies between mental and actual movement durations. This is notably the case when some participants underestimate and others overestimate mental or actual movement durations. Therefore, in order to provide a more complete analysis of mental performance, we also calculated the absolute index of mental/actual performance (aIP); that is the absolute difference between the average duration of mental movements (*D_M_* in the formula; n = 10) and the average duration of actual movements (*D_A_* in the formula; n = 10):
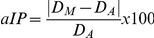



We verified that aIP showed normal distribution (Shapiro-Wilk test) and then performed ANOVA with *group* (control, patients) as between-subjects factor and *motor task* (two walking and one drawing) as within-subjects factors. For all the statistical analyses, the level of significance was fixed at *P*<0.05 and *post-hoc* comparisons were performed by means of *Scheffé tests.*

## Results

### Actual and mental movement durations

**Figure 2:**
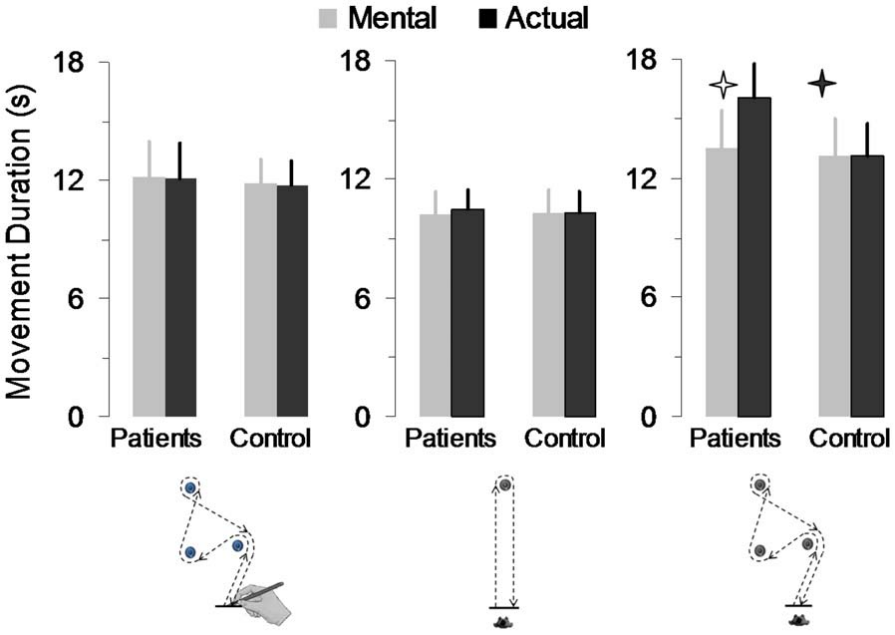
Average duration (+SD) of actual and mental movements for the two groups in the three motor tasks. The white asterisk shows significant differences between actual and mental movements, while the black asterisk shows significant differences between patients and healthy adults for the actual movements.

Fig. 2 shows average duration (+SD) of actual and mental movements for the two groups in the three motor tasks. ANOVA showed a main effect of *task* (F_2,44_ = 19.20, P<0.001) and a main effect of *execution mode* (F_1,22_ = 8.07, P<0.001). Post-hoc analysis revealed that movement durations significantly differed between the three tasks (in all cases, P<0.001; on average: 10.32±1.20 s for the 1^st^ walking task, 11.95±2.74 s for the drawing task, and 13.66±2.56 s for the 2^nd^ walking task). Furthermore, mental durations were significantly shorter than actual durations (on average: 11.77±2.54 s and 12.19±2.75 s). There was also an interaction effect between *group* and *execution mode* (F_1,22_ = 9.26, P<0.001). Post-hoc analysis revealed that actual and mental movement durations significantly differed in the group of patients (P = 0.005), but not in the control group (P = 0.99). ANOVA also showed an interaction effect between *group*, *task* and *execution mode* (F_2,44_ = 15.81, P<0.0001). Post-hoc analysis revealed a significant difference between actual and mental durations in the group of patients for the 2^nd^ walking task (P<0.0001), but not for the drawing task (P = 0.88) or the 1^st^ walking task (P = 0.99). In the control group actual and mental movement durations were equivalent (P = 0.98, for the drawing task; P = 0.99, for the 1^st^ walking task; P = 0.98 for the 2^nd^ walking task). Furthermore, actual durations of the 2^nd^ walking task were longer for group of patients than the control group (P = 0.03).

### Index of Performance (IP)

**Figure 3:**
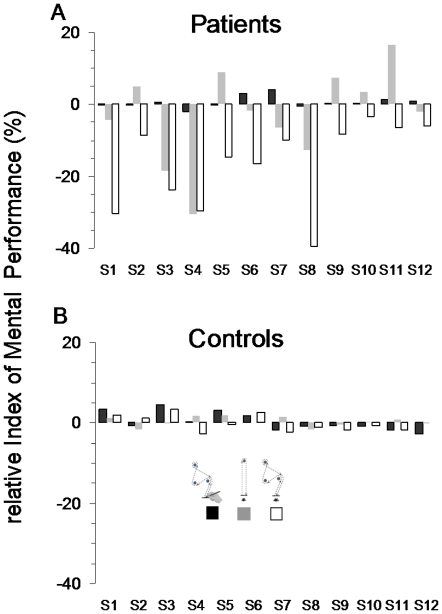
Individual average values of the relative index of mental performance (rIMP) for all the participants in the three motor tasks. (A) group of patients; (B) control group.

Fig. 3 shows the average (+SD) rIP for all participants in the three motor tasks. Positive and negative values indicate whether participants overestimate or underestimate mental movement durations with respect to their actual counterparts. Patients showed good performance for the drawing task, i.e., high temporal similarities between mental and actual movements. Note that individual rIP ranged between −1.95% and 4.26% (see Fig. 3A, black histograms). However, their performance declined during the first walking task; individual rIMP ranged between −23.37% and 19.19% (see Fig. 3A, grey histograms). Patients' performance severely deteriorated during the second walking task. All patients underestimated the duration of actual movements in the second walking task; individual rIP ranged between −3.27% and −40% (see Fig. 3A, white histograms). Participants of the control group had small rIP in the three motor tasks (individual rIP ranged between −2.71% and 4.63%), indicating that mental movement durations were very similar to actual movement durations. Healthy participants did not show any particular tendency to overestimate or underestimate the duration of actual movements.

**Figure 4:**
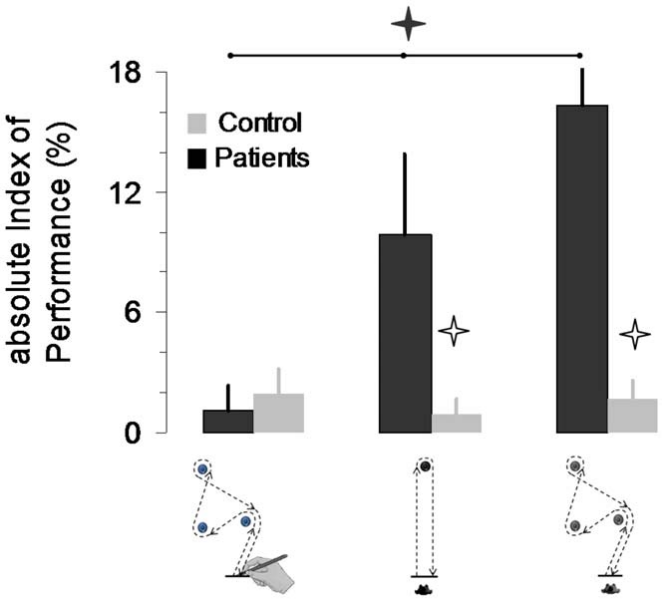
Average values (+SD) of the absolute index of mental performance (aIMP) for the two groups in the three motor tasks. The asterisks show significant differences between actual and mental movements, while the black line shows significant differences between patients and healthy adults for the three motor tasks.

Fig. 4 shows the average values (+SD) of the aIP for the two groups in the three motor tasks. The aIP was low and stable across the motor tasks for the control group, while it increased progressively according to the motor task for the group of patients. ANOVA revealed an interaction effect between *group* and *task* (F_2,44_ = 10.62, P<0.001). *Post-hoc* analysis showed that the aIP of the control group did not vary according to the motor task (P = 0.95, for the drawing versus the 1^st^ walking task; P = 0.97, for the drawing versus the 2^nd^ walking task; P = 0.91, for the 1^st^ versus the 2^nd^ walking task). On the contrary, the aIP of the group of patients significantly varied according to the motor task (P = 0.004, for the drawing versus the 1^st^ walking task; P = 0.0001, for the drawing versus the 2^nd^ walking task; P = 0.002, for the 1^st^ versus the 2^nd^ walking task). The aIP of the control group and the aIP of the group of patients significantly differed for the 1^st^ (P = 0.021) and the 2^nd^ walking task (P = 0.0002), but not for the drawing task (P = 0.94).

**Figure 5:**
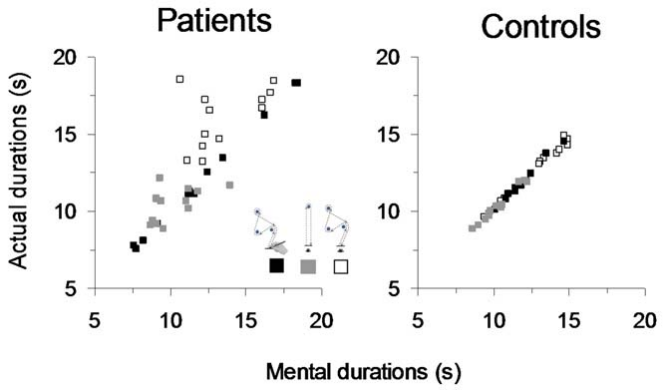
Average duration of mental movements is plotted versus the average duration of actual movements for each motor task separately. Each mark is the average value of 10 mental and 10 actual trials. A temporal dissociation between actual and mental movements is noticeable for the patients group for the two walking tasks.

In Fig. 5, average durations of mental movements are plotted across average durations of actual movements for the three motor tasks and the two groups separately. Correlations between mental and actual movement durations were lower for the group of patients compared to the control group for the 1^st^ walking task (respectively, R^2^ = 0.30, P = 0.07 and R^2^ = 0.98, P< 0.0001) and the 2^nd^ walking task (respectively, R^2^ = 0.24, P = 0.1 and R^2^ = 0.98, P<0.0001) task, but not for the drawing task (for both groups R^2^ = 0.99, P<0.0001).

## Discussion

In the current study, we investigated to what extent bilateral vestibular impairment affects action representation. Patients with bilateral vestibular damage and healthy adults actually performed and mentally simulated walking and drawing. For the control group, there was an isochrony between mental and actual movements for the three tasks. For the group of patients, we found significant temporal discrepancies between actual and mental walking movements. Conversely, drawing actual and drawing mental durations were similar. This result denotes an inconsistency between action representation and action execution following vestibular damage, which is specific to walking movements, and emphasizes the role of the vestibular system upon mental states of actions.

### Internal models of action and mental movements

We found roughly excellent timing correspondences between actual and mental movements in the group of healthy participants. These findings broaden those of previous studies, which have also argued in favour of similar spatiotemporal features between movement production and its mental replication [8], [9], [14]. We have previously reported that the involvement of internal predictive models in both actual and mental movements explains their robust isochrony [8], [38]. Internal forward models are neural networks that simulate the dynamic behaviour of the body and its interaction with the environment. During actual movements, the internal forward model relates the sensory signals of the actual state of the body (e.g. position, velocity) to the prepared motor commands and predicts the future states of the body [16]. During mental movements, motor commands are blocked before reaching the muscle level, i.e. no movement occurs. However, a copy of these motor commands is available to the forward model, which can predict the future states of the body. As a rule, motor prediction is optimal during feedforward motor control, namely, for a movement that does not require on-line feedback regulation [8], [36]. In this case, the output of the forward internal model (in our case, movement timing) is precise and equivalent for actual and mental movements. The involvement of internal predictive models may explain why mental training in healthy adults improves motor performance [15], [17], [39], [40], [41], [42]. Mental practice is increasingly used in motor rehabilitation [43], [44], [45] and has been proven to be either beneficial by itself or in addition to physical practice for patients with neurological deficits.

### Internal models of actions in patients with vestibular loss

The temporal discrepancy between actual and mental movements in the group of patients indirectly suggests that action representation declines after vestibular damage. Patients were unable to mentally replicate with temporal precision their locomotion; mental times were significantly shorter than actual ones. Interestingly, the decline in motor prediction was proportional to the task difficulty (compare the 1^st^ with the 2^nd^ walking task). These results extend those of previous studies which reported that bilateral vestibular loss, caloric and galvanic vestibular stimulation, as well as microgravity strongly affect visual imagery [28], [29], [30], [31]. As a conclusion, vestibular information seems to be important for mental events, such as visual and motor imagery, as well as mental transformations of objects and body segments.

The question is why there are temporal inconsistencies between action representation and action execution in vestibular patients. We think locomotion patterns reorganization after vestibular damage influences action representation. Successful gait requires continuous adaptation of inter-segmental coordinative patterns and of spatial orientation. Central processing of vestibular information is essential for the control of locomotion. This is supported by several data in the literature, which reported that vestibular signals are critical for the control of posture and gait [46], [47], [48], [49], [50]. Furthermore, when visual references are lacking, loss of vestibular inputs induces an erroneous perception of self-motion [51], self-orientation in space [52] and deviations from the walking direction [34]. Vestibular patients cannot use vestibular information to control (feedforward control process) their gait. Consequently, they rely on visual and proprioceptive feedback to estimate the consequences of their motor commands and to evaluate their body state. This feedback control process inevitably reduces walking speed; especially, when the motor task is very demanding. In our study, such a control strategy can be supported by two findings: (i) in the second walking task, actual movements of patients were slower than those of the control group, (ii) the patients orally reported that decreasing walking speed is an efficient strategy that allowed them to better control their gait. Therefore, for vestibular patients sensory information from the moving limbs strongly supplements and regulates central neural drives, and defines the ultimate determinants of the gait pattern, including movement timing.

During mental movements, sensory information from the periphery is lacking, and the predictions of the internal forward model are based on efferent copies that are not corrected by sensory feedback, as it is the case during actual locomotion. As a consequence, mental movement simulation is inaccurate and movement durations are shorter than actual ones. This is not the case in healthy adults, because their motor commands were overall accurate (feedforward control) and motor prediction, based on accurate efferent copies, provide similar temporal features during both actual and mental movements. Note that we can exclude a general decline in mental imagery process, which would mean a general impairment of internal forward models, because patients with vestibular loss were able to accurately simulate drawing movements. Therefore, discrepancy between actual and mental movements in vestibular patients is task-dependent and reflects the specific influence of vestibular system on action representation.

### Comparison with neurological deficits of central origin

Although our experimental data strongly suggest that action execution and its mental replication differs in vestibular patients, the central origins of this divergence are unknown. Current results are in agreement with previous studies which reported faster imagined movements compared to their actual counterparts in patients with lesions localised in the parietal cortex [18], [19]. Note that this is not the case after damages in the motor cortex [18], [19] or the cerebellum [20], which cause slowing in both actual and imagined movements. Therefore, we speculate that the lack of vestibular information in the parietal cortex, a brain area involved in the generation of sensorimotor predictions [18], [19], causes deficits in mental imagery process. This premise is corroborated by previous studies [29], which showed that mental rotation, task that activates posterior parietal cortex, was impaired during caloric vestibular stimulation.

### Non-specific effects of vestibular damage on mental actions

Two alternative explanations of our results must be excluded before concluding positively about the influence of vestibular influence on action representation. First, one could attribute the temporal dissimilarities between actual and mental movements recorded in the present study to the sensation of effort, which may be greater in vestibular patients. Previous studies reported that when subjects imagined walking [53] or pointing with an additional load [54], they significantly overestimated movement duration. It was hypothesised that the additional load was interpreted as an increase in movement duration. Sense of effort could not explain our findings, because vestibular patients imagined walking faster and not slower. Second, there is a well-known effect of the duration of the task on the accuracy of mental movements: for short durations mental movements are overestimated, while for long durations they are underestimated [11]. We consider that a *duration effect* cannot account for our findings. Indeed, such an effect should also be observed in the control group. In addition, as the drawing task was longer than the first walking task in the group of patients, one should expect a greater underestimation for the former compared to the latter. Contrariwise, there was isochrony between actual and mental drawing movements.

### Study limitations

In our study, we mainly recorded the timing of actual and mental movements. Although this method has consistently provided robust results [9], [12], [36], [37], it has limitations because time is one of the main features of mental actions. We are persuaded that current results could be further strengthening by physiological measurements [7], [55]. Notably, the recordings of skin resistance, heart rate and respiratory frequency could have provided us with valuable information regarding the degree of the patients' involvement in the motor imagery process. Such measurements, however, were not possible in the current study due to time imperatives of the group of patients.

### Clinical implications

The finding that in patients with bilateral vestibular loss mental representation of an action differs from its normal execution is of great clinical interest. This discrepancy implies that vestibular patients, at least those tested in our study, have not integrated (even after 5 years of vestibular loss) their vestibular deficit in action representation. This could suggest that during action planning vestibular patients may overestimate the capacity of their motor system (imaging faster, executing slower) with harmful consequences for their health. For instance, if a vestibular patient has to climb a staircase and he/she imagines the action faster than can be performed, he/she will prepare motor commands that challenge the state of the motor system. This will increase the probability of falling. We propose that physical therapists take into consideration the discrepancy between mental and actual movements in vestibular patients when proposing physical or mental exercise to improve their equilibrium. As mental practise could be beneficial for compensating vestibular deficits [56], patients should also be explicitly informed about this inconsistency in order to adapt, as far as possible, their motor planning.
